# Gut microbiota signature in treatment-naïve attention-deficit/hyperactivity disorder

**DOI:** 10.1038/s41398-021-01504-6

**Published:** 2021-07-08

**Authors:** Vanesa Richarte, Cristina Sánchez-Mora, Montserrat Corrales, Christian Fadeuilhe, Laura Vilar-Ribó, Lorena Arribas, Estela Garcia, Silvia Karina Rosales-Ortiz, Alejandro Arias-Vasquez, María Soler-Artigas, Marta Ribasés, Josep Antoni Ramos-Quiroga

**Affiliations:** 1 Department of Psychiatry, Mental Health and Addictions, Hospital Universitari Vall d’Hebron, Barcelona, Catalonia Spain; 2 Biomedical Network Research Centre on Mental Health (CIBERSAM), Instituto de Salud Carlos III, Madrid, Spain; 3 Department of Psychiatry and Legal Medicine, Universitat Autònoma de Barcelona, Barcelona, Catalonia Spain; 4 Psychiatric Genetics Unit, Group of Psychiatry, Mental Health and Addiction, Vall d’Hebron Research Institute (VHIR), Universitat Autònoma de Barcelona, Barcelona, Catalonia Spain; 5 Department of Biomedicine, University of Barcelona, Barcelona, Spain; 6 Department of Human Genetics, Donders Institute for Brain, Cognition and Behaviour, Radboud University Medical Center, 6525 GA Nijmegen, The Netherlands; 7 Department of Psychiatry, Donders Institute for Brain, Cognition and Behaviour, Radboud University Medical Center, 6525 GA Nijmegen, The Netherlands; 8 Department of Genetics, Microbiology & Statistics, University of Barcelona, Barcelona, Spain

**Keywords:** Scientific community, ADHD

## Abstract

Compelling evidence supports alterations in gut microbial diversity, bacterial composition, and/or relative abundance of several bacterial taxa in attention-deficit/hyperactivity disorder (ADHD). However, findings for ADHD are inconsistent among studies, and specific gut microbiome signatures for the disorder remain unknown. Given that previous studies have mainly focused on the pediatric form of the disorder and involved small sample sizes, we conducted the largest study to date to compare the gastrointestinal microbiome composition in 100 medication-naïve adults with ADHD and 100 sex-matched healthy controls. We found evidence that ADHD subjects have differences in the relative abundance of several microbial taxa. At the family level, our data support a lower relative abundance of Gracilibacteraceae and higher levels of Selenomonadaceae and Veillonellaceae in adults with ADHD. In addition, the ADHD group showed higher levels of *Dialister* and *Megamonas* and lower abundance of *Anaerotaenia and Gracilibacter* at the genus level. All four selected genera explained 15% of the variance of ADHD, and this microbial signature achieved an overall sensitivity of 74% and a specificity of 71% for distinguishing between ADHD patients and healthy controls. We also tested whether the selected genera correlate with age, body mass index (BMI), or scores of the ADHD rating scale but found no evidence of correlation between genera relative abundance and any of the selected traits. These results are in line with recent studies supporting gut microbiome alterations in neurodevelopment disorders, but further studies are needed to elucidate the role of the gut microbiota on the ADHD across the lifespan and its contribution to the persistence of the disorder from childhood to adulthood.

## Introduction

Attention-deficit/hyperactivity disorder (ADHD) is a neurodevelopmental disorder characterized by a persistent pattern of symptoms of inattention, hyperactivity, and impulsivity, resulting in dysfunction in two or more areas of an individual’s life [1,2,]. ADHD is associated with deterioration in the social, family, academic, and/or occupational functioning of the affected subjects and has a high impact at the socioeconomic level [3].

The prevalence of ADHD in children is approximately 5.3%, and of these, 50−70% will still show symptoms in adulthood [4]. The etiology is complex and multifactorial, with an average heritability of 74% [5]. Through the largest meta-analyses of genome-wide association studies performed so far, the first genome-wide significant loci for ADHD were identified [6,7,]. Evidence for a strong genetic component of common variants in the polygenic architecture of ADHD was found, with an SNP-based heritability of 22% [6]. Given that the large proportion of heritability still needs to be explained, these data also suggest that other mechanisms may provide a means for integrating the effects of genetic and environmental risk factors and explaining additional phenotypic variance in ADHD. Among such factors, compelling evidence supports a possible role for the gut microbiome in ADHD.

The gut microbiome is essential for health and plays a role in the bidirectional regulation of the brain-gut axis. Microorganisms influence the brain through their ability to produce and modify many metabolic, immunological, and neurochemical factors in the gut that ultimately impact the central nervous system [8–10]; in turn, brain activity also impacts the gut microbiota composition [11,12,]. The gut microbiota influences gut barrier integrity and produce neuroactive compounds such as neurotransmitters, amino acids, and microbial metabolites, including short-chain fatty acids [10,13,]. These metabolites can interact with the host immune system, act on the central nervous system by regulating gene expression, epigenetics and neuroplasticity and affect local neuronal cells and afferent pathways that signal directly to the brain [13]. This dynamic bidirectional communication between the gut microbiota and the central nervous system influences brain function, cognition, and behavior and highlights the fact that gut microbiota imbalance may contribute to the pathophysiology of neurodevelopmental disorders and mental health outcomes.

Consistently, an increasing number of studies have shown gut microbiome alterations in neurodevelopmental disorders [14–19]. For ADHD, an increasing number of studies have reported that the gut microbial diversity, bacterial composition, and/or relative abundance of several bacterial taxa differ between patients and healthy controls [20–28]. Although not confirmed by others [20,22,27,], some studies found differences in microbiota alpha [23,28,] or beta diversity [23,24,] in ADHD. For example, Prehn-Kristensen et al. observed decreased alpha diversity in ADHD patients and differences in beta diversity between patients and controls [23]. Wang et al. [28] also reported differences in alpha diversity in ADHD, and Szopinska-Tokov found a significant reduction in beta diversity in patients with ADHD [24,28,]. When focusing on specific taxonomic groups, Aarts et al. reported a nominal increase in *Bifidobacterium* in individuals with ADHD, changes that were associated with a significantly enhanced predicted synthesis of the dopamine precursor phenylalanine [22]. Similarly, Jiang et al reported decreased amounts of the genera *Dialister, Lachnoclostridium, Sutterella*, and *Faecalibacterium* in treatment-naïve children with ADHD compared with healthy controls and a negative association between the abundance of the last taxonomic group and parental reports of ADHD symptoms [27]. These results were consistent with those from a recent study by Wan et al., which also detected a reduced relative abundance of *Faecalibacterium*, as well as higher amounts of *Odoribacter* and *Enterococcus*, in ADHD patients [20]. Moreover, Prehn-Kristensen observed distinct abundance in different microbial taxa, including increased *Neisseria* and decreased *Prevotella* and *Parabacteroides* in ADHD subjects [23]. Wang et al. compared the fecal microbiota composition between medication-naïve children with ADHD and healthy controls and found *Fusobacterium* genus as a marker for ADHD as well as enrichment of *Lactobacillus* in the control group [28]. Finally, a recent study conducted by Szopinska-Tokov et al. revealed an association between the relative abundance of the *Ruminococcaceae _UCG_004* genus and ADHD inattention symptoms [24]. All these previous studies, however, considered small sample sizes (from 14 to 51 ADHD patients), mainly focused on the childhood/adolescent form of the disorder, and showed no overlap or lack of concordance between findings.

Additionally, clinical evidence shows that probiotic intervention in early life may improve later outcomes and reduce the risk of neuropsychiatric disorders [29], and mice colonized by microbiota from subjects with ADHD displayed altered microbial composition and behavioral and brain abnormalities compared with mice transplanted with the microbiota from individuals without ADHD [30]. These data further support that the gut microbiome composition may influence brain function and behavior and play a role in the disorder [30–33].

Considering this background, we performed the largest characterization of the gastrointestinal microbiome composition in 100 medication-naïve adults with ADHD and 100 sex-matched healthy controls and assessed differences in the microbiota composition between both groups and whether such differences were associated with ADHD clinical symptoms.

## Materials and methods

### Participants and clinical assessment

The clinical sample consisted of 100 adult medication-naïve ADHD subjects (DSM-5 criteria) who were referred to an ADHD program from primary care centers and adult community mental health services. All subjects were evaluated and recruited prospectively from a restricted geographic area of Catalonia (Spain) in a specialized outpatient program for adult ADHD and by a single clinical group at Hospital Universitari Vall d’Hebron of Barcelona (Spain). A description of the sample is provided in Supplementary Table 1.

The clinical assessment consisted of structured interviews and self-report questionnaires in two different steps: (i) ADHD diagnosis was based on the results of the Structured Diagnostic Interview for Adult ADHD (DIVA 2.0) [34] by a psychiatrist; (ii) the severity of ADHD symptoms and levels of impairment and comorbid disorders were assessed by a psychologist. In this part of the evaluation, the following scales and questionnaires were used: the ADHD Rating Scale (ADHD-RS), the Clinical Global Impression (CGI), the Wender Utah Rating Scale (WURS), the Sheehan Disability Inventory (SDS), and the Structured Clinical Interview for DSM-IV Axis I and II Disorders (SCID-I and SCID-II). Afterward, the psychiatrist and psychologist integrated the clinical information and self-reports for valid assessment of symptoms and impairments. In case of discordance between the different raters for ADHD symptoms or inconsistencies between the reporters in responses to items measuring similar symptoms, clinician-identified symptoms on the DIVA 2.0 prevailed. Clinical information was reordered at the moment of inclusion, at which time the stool specimen was also collected. Exclusion criteria were as follows: an intelligence quotient less than 70; lifelong or current history of mood, psychotic, anxiety, substance abuse, and personality disorders; pervasive developmental disorders; a history or the current presence of a condition or illness, including neurologic, metabolic, cardiac, liver, kidney, or respiratory disease; a chronic medication of any kind; birth weight ≤ 1.5 kg; and other neurological or systemic disorders that might explain ADHD symptoms.

The control sample consisted of 100 unrelated healthy donors matched by sex and ethnicity with the clinical group. The exclusion criteria were ADHD symptomatology according to the Adult Self-Report Scale A.S.R. S v1.1. and any prior or current psychiatric comorbidity.

All subjects reported European ancestry, which was confirmed through principal component analysis (PCA) using genetic data. Exclusion criteria for all participants included treatment with antibiotics or probiotics up to before stool collection.

The study was approved by the Clinical Research Ethics Committee (CREC) of Hospital Universitari Vall d’Hebron. All methods were performed in accordance with the relevant guidelines and regulations, and written informed consent was obtained from all subjects before inclusion. None of the participants received any financial compensation.

### Sample collection and DNA isolation

Human fecal samples were collected at home, stabilized with the OMNIgene·GUT (OM-200) (DNA Genotek Inc.) kit, and then transported to the laboratory. The samples were aliquoted into 1.5-ml tubes and stored at −80 °C. Microbial DNA was purified from 200 mg of each homogenized fecal sample using the QIAamp^®^ PowerFecal® DNA extraction kit (QIAgen, Hilden, Germany). The isolated DNA was quantified using PicoGreen™ dsDNA Assay Kit [35].

### Library preparation and Illumina sequencing

The V3−V4 hypervariable region of the bacterial 16S rRNA gene was amplified for microbiome composition profiling. DNA library construction was performed following the manufacturer’s instructions (Illumina). We used the same workflow as described elsewhere [36] to perform cluster generation, template hybridization, isothermal amplification, linearization, blocking and denaturation, and hybridization of the sequencing primers. Briefly, the V3−V4 region was amplified using key-tagged eubacterial primers 5′CCTACGGGNGGCWGCAG3′ and 5′GACTACHVGGGTATCTAATCC3′, and 300-nt paired-end amplicons were subsequently sequenced in two different rounds using the Illumina MiSeq platform. The raw Illumina paired-end reads were merged considering an overlap length > 70 bp with the PEAR software v. 0.9.1, providing a single FASTQ file for each of the samples [37]. High-quality reads were extracted by applying a minimum Phred score of 20 (Q20, 99% based call accuracy). After primer sequences trimming, reads without both primer sequences or with less than 200 bp were discarded with Cutadapt v.1.8.1 [38]. Chimeric sequences were removed using the UCHIME software [39]. After quality control filtering, we obtained 14.7 million high-quality sequences with 45 063−216 059 reads per sample from a total of 200 fecal samples. The raw and clean number of sequences, mean length, total mega bases sequenced, and mean quality per sample can be found in Supplementary Table 2. The remaining reads were clustered into operational taxonomic units (OTUs), in which unique sequences with a relative abundance above 0.1% were clustered into OTUs based on 97% sequence similarity [40] using the CD-HIT package [41] and the BLAST search against the NCBI 16S rRNA reference database (September 2019) with bastn v.2.10.0+. Taxonomic groups (phylum, family, and genus) were assigned with a Python script developed by ADM-BIOPOLIS (Paterna, Valencia, Spain). To remove genera with absent or low prevalence, the OTU table was filtered at the genus level. OTUs with nonzero values in less than 10% of the samples were removed. OUT counts were normalized by rarefaction with the phyloseq R package according to Weiss et al. [42].

### Statistical analysis

Alpha diversity (within-sample diversity) was calculated on rarefied data with the Richness, Simpson and Shannon diversity indices and compared between individuals with ADHD and controls using the vegan R package (https://github.com/vegandevs/vegan). Beta diversity (between-sample diversity) was calculated by weighted and unweighted UniFrac and Bray Curtis distances, as represented by two-dimensional principal coordinates analysis (PCoA) plots, and compared between groups by permutation multivariate analysis of variance (PERMANOVA) using the phyloseq R package [43]. The local contribution to beta diversity (LCBD) test was applied to evaluate the contribution of each sample to the diversity between the groups using the adespatial R package (https://github.com/sdray/adespatial). Canonical correspondence analysis (CCA), a multivariate constrained ordination method, on rarefied OTUs was performed and significance regarding the microbial community composition between groups was assessed by permutational multivariate analysis of variance (ADONIS) using the vegan R package (https://github.com/vegandevs/vegan).

Differential abundance comparisons between groups were assessed in taxonomic groups showing an average of normalized counts (baseMean) > 10 using the DESeq2 and randomForest R packages for the classification, rfUtilities to estimate the significance of the classification and rfPermute to evaluate the significance of specific taxa, with 1000 permutations. All comparisons were performed at the phylum, family, and genus levels. Any unknown taxonomic level was assigned to the next highest known taxonomic rank.

Genera showing significant differences in relative abundance between ADHD cases and controls after multiple comparison corrections in DeSeq2 and the random forest comparisons were considered for downstream analyses. Multiple logistic regression models were applied to test the association between ADHD and all selected genera while adjusting for age, sex, and body mass index (BMI). Adjusted Pseudo-R2 was calculated with the McFaddenAdj method and the DescTools R package (https://github.com/AndriSignorell/DescTools); sensitivity and specificity were calculated with the caret R package (https://github.com/topepo/caret/). A likelihood ratio test with the lmtest R package (https://cran.r-project.org/web/packages/lmtest/) was employed to assess whether the inclusion of selected genera in the multiple logistic regression model fits the data significantly better than the model including only age, sex, and BMI. In the first model, we considered affectation status as dependent variable and age, sex and BMI as independent variables (ADHD 〜 age + sex + BMI); in the second model, we included selected taxa as independent variables (ADHD 〜 age + sex + BMI + *Megamonas* + *Anaerotaenia* + *Gracilibacter* + *Dialister)*. Spearman correlation tests were used to assess correlations between selected genera, age, BMI, and inattention and hyperactive/impulsivity subscale scores or total scores of the ADHD rating scale.

## Results

Bacterial composition based on 16S rRNA sequencing was available for 100 adult ADHD cases and 100 controls. No differences in intestinal microbial alpha diversity (microbial community richness and evenness) were found between ADHD cases and controls when measured by three different indices (Richness, Simpson, or Shannon indices; Supplementary Fig. 1). Beta diversity (between-sample community dissimilarity) according to weighted and unweighted UniFrac distances as well as the Bray-Curtis dissimilarity index showed no differences in the microbial composition between the groups (PERMANOVA P-value>0.05), with no evidence of separate clustering in PCoA representations (Supplementary Fig. 2). No significant differences in the gut microbiota composition between the ADHD and control groups were observed in the CCA either (ADONIS *P*-value = 0.31; Supplementary Fig. 3).

**Fig. 1:**
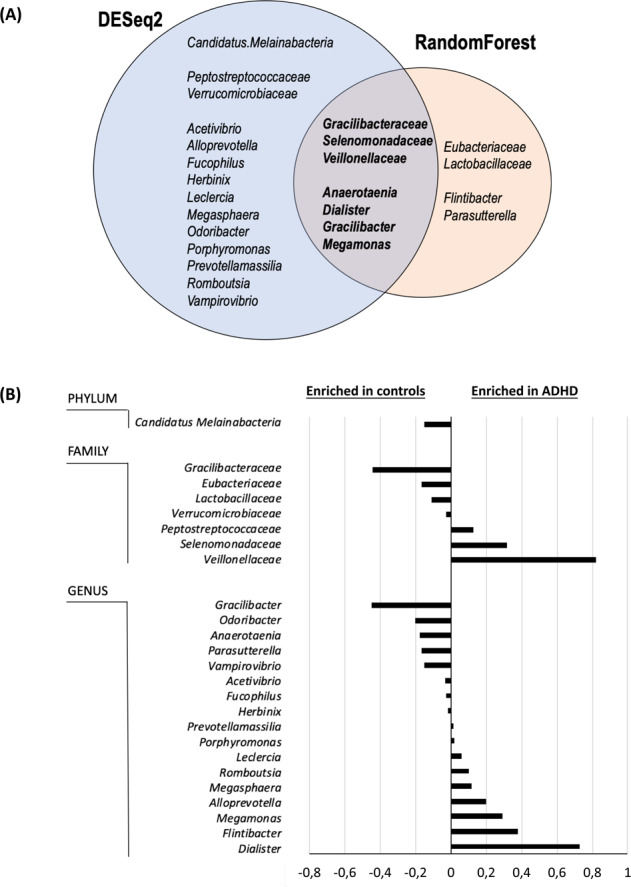
Differentially abundant taxa between ADHD cases and controls. (**A**) Differential abundance results according to two different methods, DESeq2 andrandom forests. **B** Differences in relative abundance between ADHD cases and controls for taxonomic groups surpassing multiple comparison corrections in DeSeq2 and/or random forest analyses.

Compositional analysis of samples revealed that *Bacteroidetes*, *Firmicutes*, *Proteobacteria*, *Actinobacteria*, and *Verrucomicrobia* were the most abundant phyla in our sample of 200 subjects (Supplementary Table 3), with no significant differences in relative abundance detected for any of them. When we explored the relative abundance of specific microbial taxa, however, we found evidence that several taxa differed significantly between ADHD cases and controls by two different methods, DeSeq2 and/or random forests: 1 phylum, 7 families, and 17 genera showed differential abundance (*P*_FDR_ < 0,05; DESeq2: 1 phylum, 5 families, and 15 genera; random forests: 5 families and 6 genera; Table 1, Fig. 1, Supplementary Table 4 and Supplementary Fig. 4). When combining the results of both methods, we found overlap for three families (Gracilibacteraceae, Selenomonadaceae, and Veillonellaceae) and four genera (*Anaerotaenia, Dialister, Gracilibacter, and Megamonas)* (Table 1 and Fig. 1).

**Table 1:** Summary of differential abundance results between ADHD patients and controls considering Deseq2 and random forest results.

		Relative abundance (% mean (SD)		Adjusted *P*-value	
		ADHD	Controls	DEseq2	Random forests
Phylum	*Candidatus Melainabacteria*	0.072 (0.24)	0.22 (0.76)	3.1E−03	0.11
Family	Eubacteriaceae	2.105 (1.51)	2.269 (1.35)	0.81	0.02
	**Gracilibacteraceae**	0,503 (0.85)	0,949 (1.49)	**0.035**	**0.05**
	Lactobacillaceae	0.965 (1.52)	1.077 (1.24)	0.93	0.02
	Peptostreptococcaceae	0,327 (0.55)	0,199 (0.23)	0.016	0.27
	**Selenomonadaceae**	0,387 (1.14)	0,071 (0.26)	**3.5E−07**	**0.05**
	**Veillonellaceae**	1,658 (1.90)	0,837 (1.43)	**0.012**	**9.9E−03**
	Verrucomicrobiaceae	0,036 (0.11)	0,063 (0.17)	0.012	0.73
Genus	*Acetivibrio*	0.021 (0.05)	0.056 (0.17)	6.1E−03	0.099
	*Alloprevotella*	0.380 (1.63)	0.182 (0.97)	4.4E−04	0.21
	***Anaerotaenia***	**0.072 (0.13)**	**0.248 (0.49)**	**2.3E−09**	**9.9E−03**
	***Dialister***	**1.377 (1.76)**	**0.649 (1.26)**	**0.041**	**0.02**
	*Flintibacter*	1.967 (1.46)	1.588 (1.37)	0.26	0.045
	*Fucophilus*	0.036 (0.11)	0.064 (0.17)	0.012	0.42
	***Gracilibacter***	**0.509 (0.86)**	**0.958 (1.50)**	**0.040**	**9.9E−03**
	*Herbinix*	0.024 (0.05)	0.042 (0.08)	0.024	0.24
	*Leclercia*	0.084 (0.42)	0.025 (0.12)	9.8E−03	0.30
	***Megamonas***	**0.323 (1.04)**	**0.029 (0.20)**	**3.2E−29**	**9.9E−03**
	*Megasphaera*	0.209 (0.72)	0.091 (0.42)	7.5E−20	0.80
	*Odoribacter*	0.547 (0.34)	0.751 (0.83)	0.039	0.14
	*Parasutterella*	0.751 (1.30)	1.588 (1.37)	0.70	9.9E−03
	*Porphyromonas*	0.129 (0.52)	0.110 (0.55)	6.1E−03	0.36
	*Prevotellamassilia*	0.356 (1.82)	0.340 (1.69)	6.4E−15	0.31
	*Romboutsia*	0.228 (0.52)	0.126 (0.16)	9.8E−03	0.93
	*Vampirovibrio*	0.073 (0.24)	0.225 (0.77)	2.6E−03	0.38

Differentially abundant taxa identified by both methods, DEseq2 and random forests, are shown in bold.

**Fig. 2:**
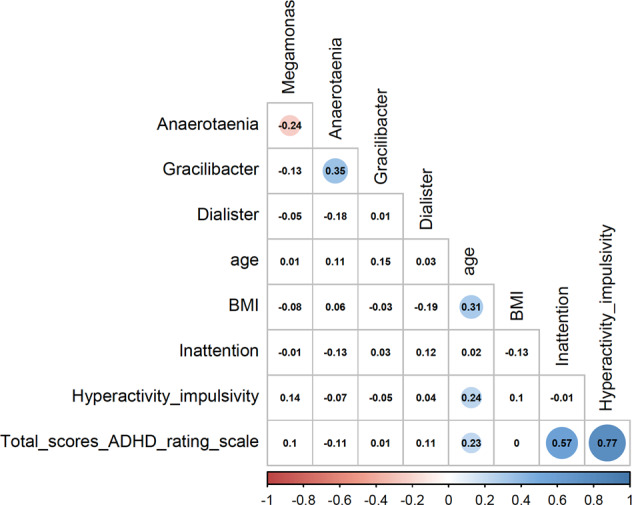
Spearman correlation between the *relative abundance of* four bacterial genera (*Anaerotaenia, Dialister, Gracilibacter,* and *Megamonas)* and age, BMI, and ADHD rating scale scores. Colored correlations are statistically significant (*P*-value < 0.05), with positive and negative correlations in blue and red, respectively. Inattention: score of the inattention subscale of the ADHD rating scale; hyperactivity_impulsivity: score of the hyperactive/impulsivity subscale of the ADHD rating scale; total: total scores of the ADHD rating scale.

For downstream analysis, we focused on genera that differed in relative abundance between ADHD and controls with both of the methods described above (*Anaerotaenia, Dialister, Gracilibacter, and Megamonas)*. When we assessed whether they correlated with each other, we found a moderate correlation between *Anaerotaenia* and *Gracilibacter* (*r* = 0.35; *P*-value = 3.6e−04), a weak correlation between *Anaerotaenia* and *Megamonas* (*r* = −0.24; *P*-value = 0.018), and no correlation between the others (Fig. 2). A model including the four genera and the covariates age, sex, and BMI explained 15% of the variance in ADHD, with significant improvement of the model which included only the covariates (*P*-value = 8.2e−07), which explained 5.9% of the variance (Supplementary Table 5). The microbial signature achieved an overall sensitivity of 74% and a specificity of 71% for the detection of individuals with ADHD versus healthy controls. We also assessed whether the selected genera correlated with age, BMI, or ADHD rating scale scores but found no evidence of correlation between relative abundance and any of the selected traits (Fig. 2).

## Discussion

To clarify the relationship between ADHD and the gut microbiome, we performed the largest study to date and compared the microbial composition between 100 medication-naïve adults with ADHD and 100 sex-matched unrelated healthy subjects. We found evidence that ADHD subjects exhibit differences in the relative abundance of several microbial taxa. At the family level, our data support a lower relative abundance of Gracilibacteraceae and higher levels of Selenomonadaceae and Veillonellaceae in adults with ADHD. In addition, the ADHD group showed higher levels of *Dialister* and *Megamonas* and lower abundances of *Anaerotaenia and Gracilibacter* at the genus level.

These results are in line with recent studies supporting gut microbiome differences in neurodevelopmental disorders. Although the mechanistic explanation for these associations remains unknown, a positive correlation between *Dialister* abundance and activity level has been described in toddlers [44]. Additionally, decreased levels of *Dialister* were found in autism spectrum disorder (ASD) patients [45,46,] or in treatment-naïve children with ADHD [27] compared with healthy controls and in ADHD individuals on medication compared with nonmedicated individuals [24]. Furthermore, multiple taxonomic groups that differed in relative abundance between ADHD cases and controls in the present study, including Selenomonadaceae, Veillonellaceae, and *Megamonas*, have previously been associated with other psychiatric conditions that often coexist with ADHD, such as ASD or depression [27, 47–51]. Given that the ADHD subjects in this study displayed no comorbid psychiatric disorders, we cannot discount a possible pleotropic effect of these taxonomic groups and that their relative abundance may explain, in part, ADHD phenotypic variability.

Although previous gut microbiome analyses on ADHD have mainly focused on pediatric samples [20,23, 26–28] and there is limited research on adults [22,24,], we focused our study on adulthood ADHD. Nevertheless, given that the gut microbiome evolves throughout the lifespan [16,52,53,], whether early-life exposure to environmental risk factors contributes to the gut microbiota and impacts neurodevelopment and mental health outcomes later in life remain to be investigated. Further longitudinal studies are warranted to provide additional information on the role of the microbiome in ADHD symptom trajectories from childhood to adulthood as well as mental health outcomes and comorbid profiles across the lifespan.

We did not detect substantial changes in alpha or beta diversity between ADHD cases and controls. The high heterogeneity in terms of age, sample size, sex, clinical characteristics, and type of controls may explain nonreplicable results and discrepancies between studies. We sex-matched ADHD cases and controls and restricted the clinical sample to ADHD medication-naïve adult subjects, which is a major strength of our study design that may allow us to identify an imbalance in the gut microbiome composition that might be neglected by broader study designs. In addition, the sample sizes of previous studies on ADHD, were relatively small; although our study may also have limited statistical power to estimate the magnitude of the differences identified in microbial relative abundance, we assessed the largest sample size considered thus far. The results, however, need to be interpreted with caution given that we selected genera of interest and estimated the variance in ADHD explained by these taxa as well as the sensitivity and specificity of the regression model using the same dataset, which may have led to overfitting and further support the use of independent datasets to obtain more accurate estimates.

Microbiome composition is strongly influenced by environmental factors such as diet, overall health status, and medication use [52, 54–56]. The participants in this study were not on medication and had not used antibiotics or probiotics in the three months before sample collection, which may not explain the differences detected between ADHD cases and controls. Nonetheless, no other environmental exposures, including smoking, stress, dietary habits, or other lifestyle information, that may have an effect on microbiota composition were considered. For instance, animal models and population-based cross-sectional studies support an effect of nicotine or smoking status on the gut microbiome composition and the fecal metabolome [57–59]. In addition to environmental factors, consistent evidence suggests that the host genetic background impacts the composition of gut microbial communities and that genetic factors influence microbiome composition and explain a significant proportion of the variation in the gut microbiome [60–63]. Hence, further integrative studies considering multiple data sources (i.e., larger sample sizes), including environmental factors, human genetic variation, and gut microbial composition, are warranted to provide deeper insight into the mechanisms underlying the relationship between the microbiota, host genetics, and individual habits, and behavior, as well as their roles in ADHD and other neurodevelopmental disorders across the lifespan.

## Supplementary information

SupplementaryTables

SupplementaryTable_S2. Raw and clean number of sequences, mean length, total mega bases sequenced, and mean quality per sample.

Supplementary_figure1. Boxplots depicting alpha diversity at the genus level between ADHD cases and controls with (a) the Shannon, (b) Chao1 and (c) Simpson indices.

Supplementary_figure2. Beta-diversity of gut microbial communities in ADHD cases and healthy controls. Principal component analysis (PCoA) plot based on weighted and unweighted UniFrac and Bray Curtis distances for 100 ADHD cases and 100 healthy controls. Two first principal components are show.

Supplementary_figure3. Comparison of the microbiota between ADHD cases and controls with canonical correspondence analysis (CCA).

Supplementary_figure4.Volcano plot showing differential microbiome composition between 100 ADHD cases and 100 controls found with the DESeq2 method. Red dots represent significant differential abundances of species (PFDR<0.05). A positive log2-fold change determined the genera overrepresented in ADHD, and a negative log2-fold change corresponded to the genera overrepresented in the control sample.

## Data Availability

The datasets supporting the conclusions of this article are included and available online. Raw fastq data will be available upon request to the corresponding author.
