# Supplementary material for: Gut microbiota signature in treatment-naïve attention-deficit/hyperactivity disorder
Source: Transl Psychiatry. 2021 Jul 8;11:382. doi: 10.1038/s41398-021-01504-6 (PMC8266901; doi:10.1038/s41398-021-01504-6)
Supplement: Supplementary file 1 — SupplementaryTables [file 41398_2021_1504_MOESM1_ESM.docx]

**Supplementary Table 1.** Description of ADHD cases and controls included in the study

| **Variables** | **ADHD** | **Controls** |
| --- | --- | --- |
| **N** | 100 | 100 |
| **% males** | 51% | 47% |
| **Age (mean ± SD)** | 33 **±** 11 years | 30 **±** 8 years |
| **Age range** | 18 - 59 years | 19 - 43 years |
| **BMI (mean ± SD)** | 24.7 **±** 4.2 | 22.1 **±** 2.9 |
| **BMI range** | 16.8 - 30.0 | 16.9 - 38.0 |
| **ADHD clinical presentations** | | |
| **Combined** | 42% | - |
| **Predominantly Inattentive** | 54% | - |
| **Predominantly Hyperactivity/impulsivity** | 4% | - |

**Supplementary Table 3.** Relative abundance of bacterial phyla in 100 ADHD cases and 100 controls

| **Phylum** | **ADHD**  **mean (SD)** | **Controls**  **mean (SD)** | **adjusted P-value** |
| --- | --- | --- | --- |
| ***Bacteroidetes*** | 48.5 (8.7) | 47.7 (9.2) | 0.81 |
| ***Firmicutes*** | 41.5 (8.2) | 41.9 (8.3) | 0.98 |
| ***Proteobacteria*** | 6.1 (3.8) | 5.9 (3.7) | 0.89 |
| ***Actinobacteria*** | 1.3 (1.7) | 1.4 (1.6) | 0.98 |
| ***Verrucomicrobia*** | 1.3 (2.5) | 1.3 (2.5) | 0.98 |

**Supplementary Table 4.** Summary of significant differential bacterial composition results between 100 ADHD cases and 100 controls at the phylum, family and genus taxon levels using Deseq2

|  |  | **baseMean** | **log_2_FC *** | **lfcSE** | **stat** | **pvalue** | **padj** |
| --- | --- | --- | --- | --- | --- | --- | --- |
|  |  |  |  |  |  |  |  |
| *Phylum* | **Underrepresented in ADHD** | | | | | | |
|  | *Candidatus Melainabacteria* | 38.56 | -1.50 | 0.40 | 3.74 | 1.82E-04 | 3.09E-03 |
|  |  |  |  |  |  |  |  |
| *Family* | **Overrepresented in ADHD** | | | | | | |
|  | Selenomonadaceae^#^ | 104.10 | 2.52 | 0.44 | -5.75 | 9.08E-09 | 3.49E-07 |
|  | Veillonellaceae^#^ | 551.60 | 1.01 | 0.31 | -3.28 | 1.06E-03 | 1.16E-02 |
|  | Peptostreptococcaceae^#^ | 116.82 | 0.73 | 0.23 | -3.15 | 1.66E-03 | 1.59E-02 |
|  | **Underrepresented in ADHD** | | | | | | |
|  | Gracilibacteraceae | 315.69 | -0.91 | 0.32 | 2.81 | 4.97E-03 | 3.48E-02 |
|  | Verrucomicrobiaceae | 19.94 | -1.18 | 0.36 | 3.28 | 1.04E-03 | 1.16E-02 |
|  |  |  |  |  |  |  |  |
| *Genus* | **Overrepresented in ADHD** | | | | | | |
|  | *Megamonas* | 74.55 | 4.83 | 0.41 | -11.70 | 1.21E-31 | 3.22E-29 |
|  | *Megasphaera* | 13.46 | 3.44 | 0.36 | -9.64 | 5.64E-22 | 7.53E-20 |
|  | *Prevotellamassilia* | 12.51 | 2.89 | 0.35 | -8.31 | 9.58E-17 | 6.39E-15 |
|  | *Alloprevotella* | 21.27 | 1.73 | 0.40 | -4.36 | 1.32E-05 | 4.41E-04 |
|  | *Leclercia^#^* | 10.52 | 1.14 | 0.34 | -3.40 | 6.62E-04 | 9.78E-03 |
|  | *Porphyromonas* | 10.12 | 1.14 | 0.32 | -3.59 | 3.29E-04 | 6.06E-03 |
|  | *Dialister* | 436.03 | 1.09 | 0.39 | -2.78 | 5.47E-03 | 4.14E-02 |
|  | *Romboutsia^#^* | 77.16 | 0.86 | 0.26 | -3.39 | 6.96E-04 | 9.78E-03 |
|  | **Underrepresented in ADHD** | | | | | | |
|  | *Odoribacter* | 279.94 | -0.45 | 0.16 | 2.82 | 4.87E-03 | 3.94E-02 |
|  | *Herbinix* | 14.45 | -0.79 | 0.26 | 3.00 | 2.74E-03 | 2.44E-02 |
|  | *Gracilibacter* | 313.40 | -0.91 | 0.33 | 2.80 | 5.10E-03 | 4.00E-02 |
|  | *Acetivibrio#* | 14.19 | -1.01 | 0.28 | 3.59 | 3.30E-04 | 6.06E-03 |
|  | *Fucophilus* | 19.92 | -1.18 | 0.36 | 3.28 | 1.02E-03 | 1.24E-02 |
|  | *Vampirovibrio* | 38.74 | -1.55 | 0.40 | 3.85 | 1.17E-04 | 2.61E-03 |
|  | *Anaerotaenia^#^* | 69.28 | -1.78 | 0.27 | 6.59 | 4.32E-11 | 2.31E-09 |
|  |  |  | - |  |  |  |  |
|  | * log_2_FC*: log_2_ fold change; lfcSE: log_2_ fold change estandard error  # Statistically significant when adjusted by age and BMI | | | | | | |

**Supplementary Table 5.** Multiple regression models and Likelihood Ratio Test

| **Independent Variable** | **B (Standard error)** | **P-value** | **R2** |
| --- | --- | --- | --- |
| **Model 1** | | | |
| **Age** | 0.005 (0.016) | 0.75 | 0.059 |
| **Sex** | 0.26 (0.32) | 0.42 |  |
| **BMI** | 0.21 (0.05) | 4.8e-05 |  |
| **Model 2** | | | |
| **Age** | 0.008 (0.017) | 0.64 | 0.15 |
| **Sex** | 0.36 (0.35) | 0.30 |  |
| **BMI** | 0.20 (0.05) | 3.3e-04 |  |
| ***Anaerotaenia*** | -2.28 (1.07) | 0.033 |  |
| ***Dialister*** | 0.34 (0.12) | 4.5e-03 |  |
| ***Gracilibacter*** | -0.13 (0.15) | 0.40 |  |
| ***Megamonas*** | 1.53 (0.68) | 0.023 |  |
| **Likelihood Ratio Test** | Chi2=33.8; df=4; P-value=8.2e-07 | | |
