# Supplementary figures and images for: Gut microbiota signature in treatment-naïve attention-deficit/hyperactivity disorder
Source: Transl Psychiatry. 2021 Jul 8;11:382. doi: 10.1038/s41398-021-01504-6 (PMC8266901; doi:10.1038/s41398-021-01504-6)

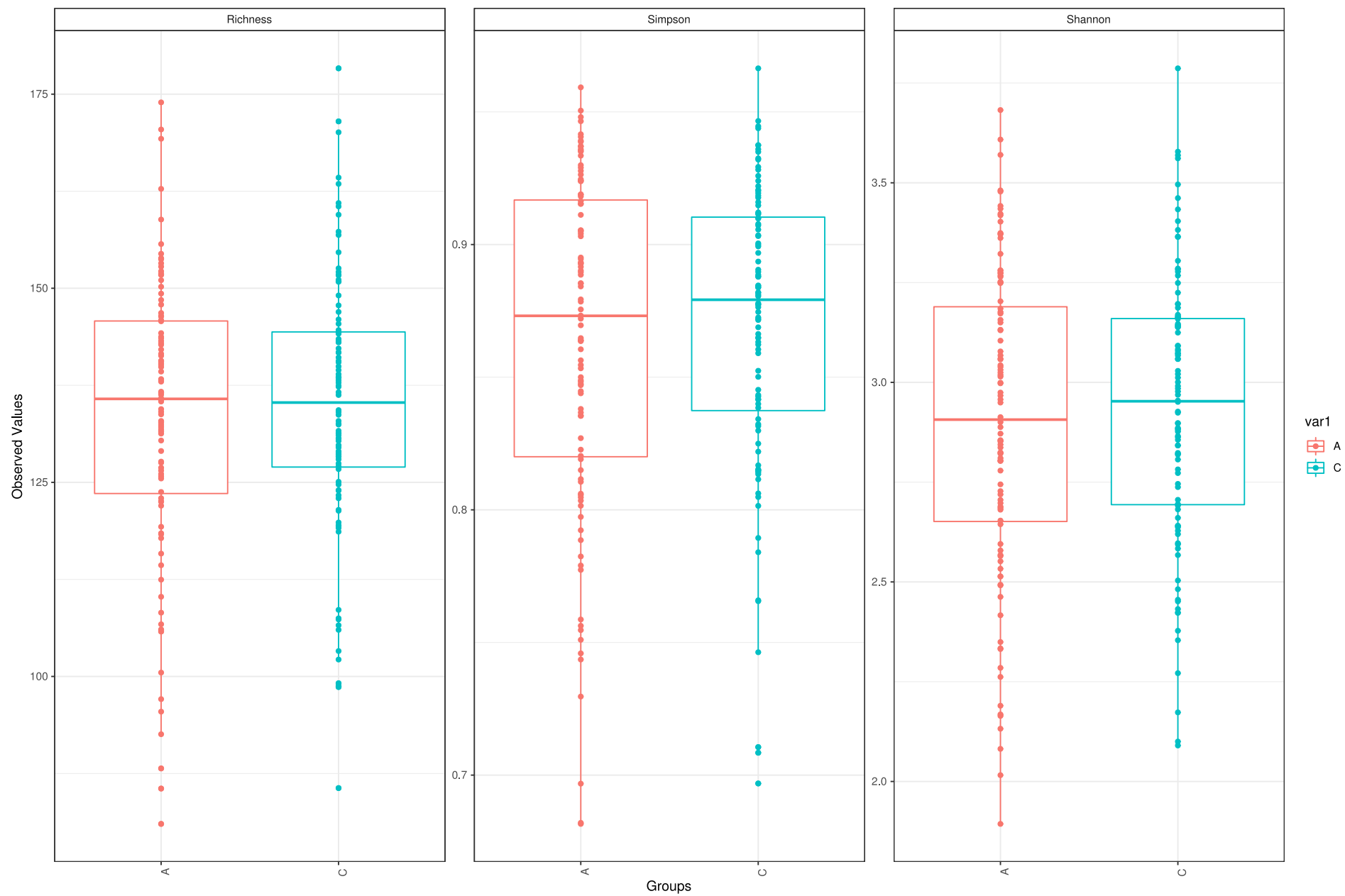

Supplement: Supplementary file 3 — Supplementary_figure1. Boxplots depicting alpha diversity at the genus level between ADHD cases and controls with (a) the Shannon, (b) Chao1 and (c) Simpson indices. [file 41398_2021_1504_MOESM3_ESM.pdf]

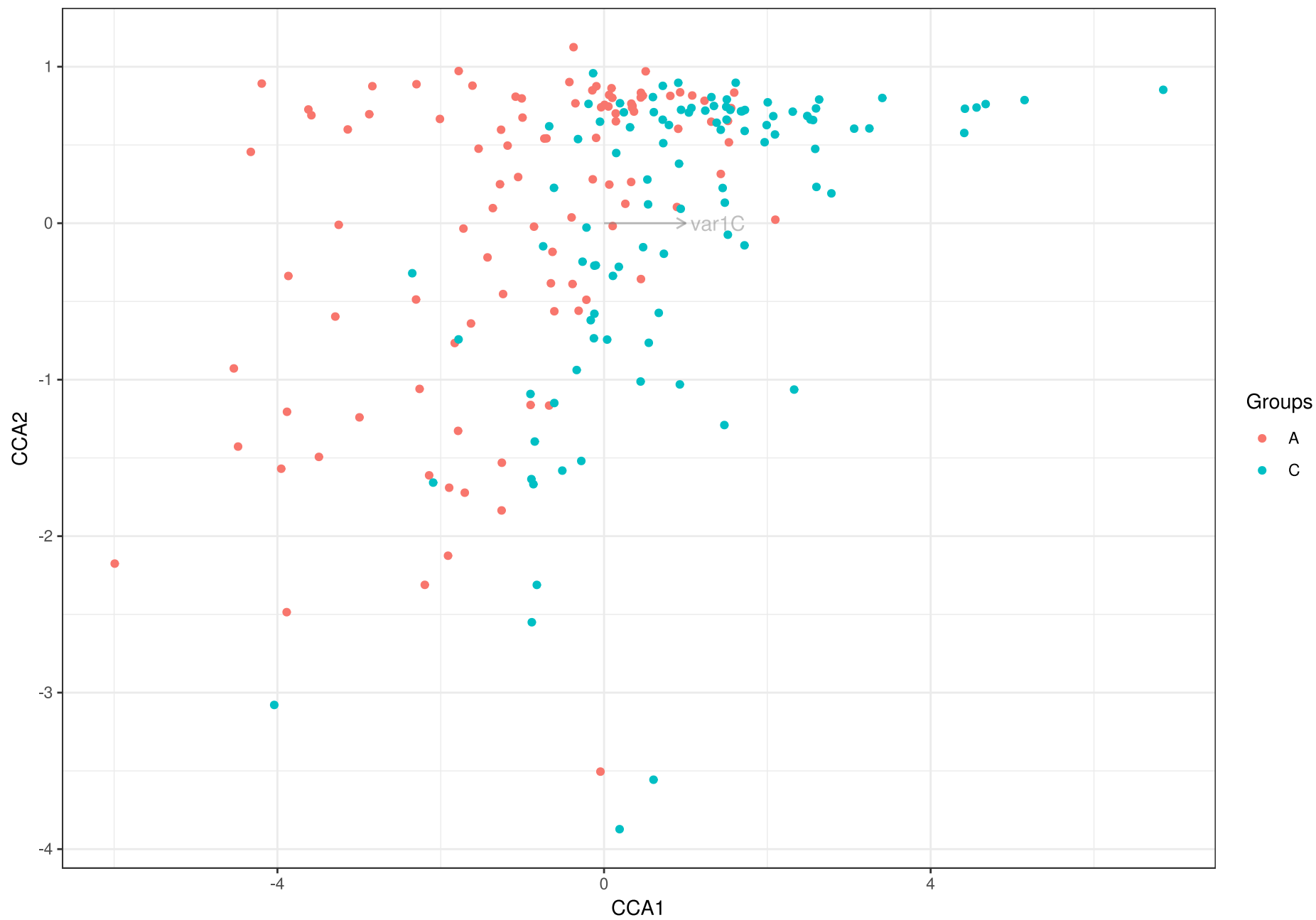

Supplement: Supplementary file 5 — Supplementary_figure3. Comparison of the microbiota between ADHD cases and controls with canonical correspondence analysis (CCA). [file 41398_2021_1504_MOESM5_ESM.pdf]

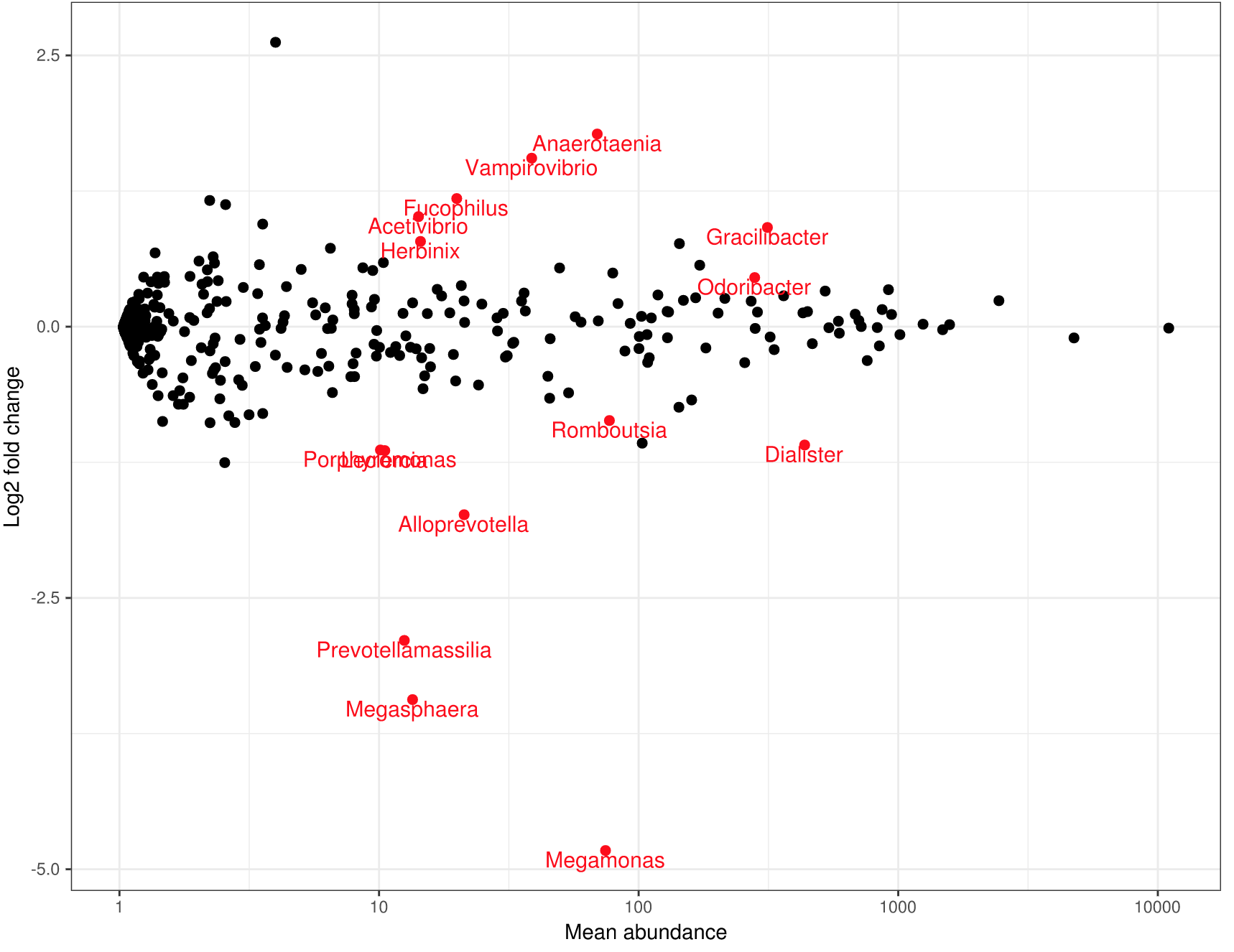

Supplement: Supplementary file 6 — Supplementary_figure4.Volcano plot showing differential microbiome composition between 100 ADHD cases and 100 controls found with the DESeq2 method. Red dots represent significant differential abundances of species (PFDR<0.05). A positive log2-fold change determined the genera overrepresented in ADHD, and a negative log2-fold change corresponded to the genera overrepresented in the control sample. [file 41398_2021_1504_MOESM6_ESM.png]
