# Supplementary material for: Gut microbiota signature in treatment-naïve attention-deficit/hyperactivity disorder
Source: Transl Psychiatry. 2021 Jul 8;11:382. doi: 10.1038/s41398-021-01504-6 (PMC8266901; doi:10.1038/s41398-021-01504-6)
Supplement: Supplementary file 4 — Supplementary_figure2. Beta-diversity of gut microbial communities in ADHD cases and healthy controls. Principal component analysis (PCoA) plot based on weighted and unweighted UniFrac and Bray Curtis distances for 100 ADHD cases and 100 healthy controls. Two first principal components are show. [file 41398_2021_1504_MOESM4_ESM.pdf]

Bray-Curtis distance

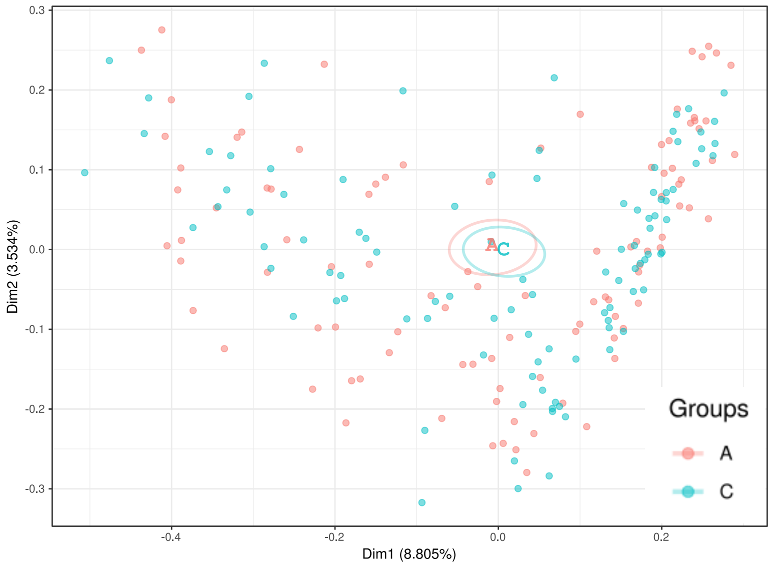

PERMANOVA
R^2=0.006
P-value = 0.316

Unweighted Unifrac distance

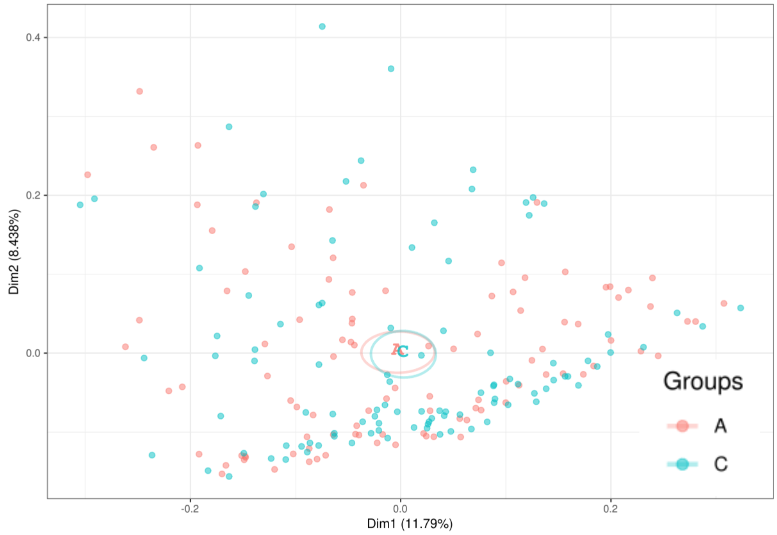

PERMANOVA
R2=0.006
P-value = 0.125

Weighted Unifrac distance

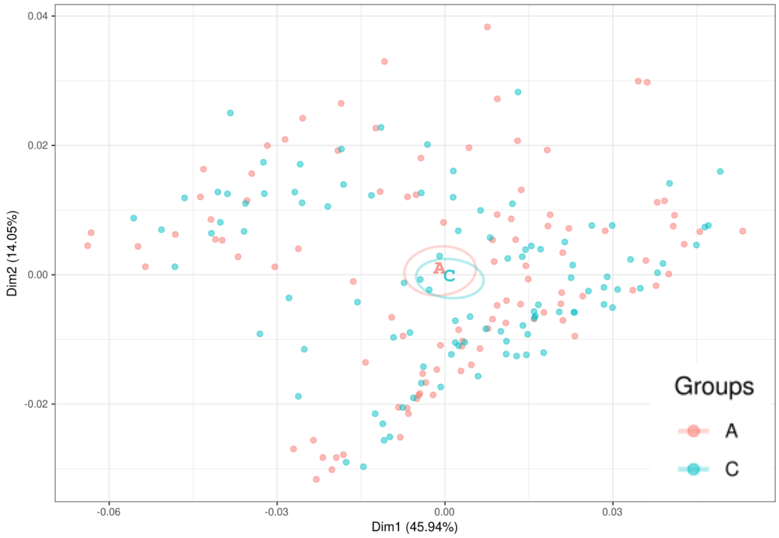

PERMANOVA
R^2=0.004
P-value = 0.54
